# Facile Synthesis of Ligand-Free Iridium Nanoparticles and Their In Vitro Biocompatibility

**DOI:** 10.1186/s11671-018-2621-3

**Published:** 2018-07-13

**Authors:** Anna L. Brown, Hayden Winter, Andrea M. Goforth, Gaurav Sahay, Conroy Sun

**Affiliations:** 1 Department of Pharmaceutical Science, Oregon State University, 2730 SW Moody Ave, Portland, OR 97201 USA; 2 Department of Chemistry, Portland State University, 1719 SW 10th Ave, Portland, OR 97201 USA; 3 Department of Biomedical Engineering, Oregon Health and Science University, 2730 SW Moody Ave, Portland, OR USA; 4 Department of Radiation Medicine, Oregon Health and Science University, 3181 SW Sam Jackson Park Road, Portland, OR USA

**Keywords:** Nanoparticle synthesis, Nanocrystals, Iridium, Surface characterization, Cellular toxicity

## Abstract

High-density inorganic nanoparticles have shown promise in medical applications that utilize radiation including X-ray imaging and as radiation dose enhancers for radiotherapy. We have developed an aqueous synthetic method to produce small (~ 2 nm) iridium nanoparticles (IrNPs) by reduction of iridium(III) chloride using a borohydride reducing agent. Unlike other solution-based synthesis methods, uniform and monodispersed IrNPs are produced without the use of surfactants or other solubilizing ligands. These nanoparticles are highly crystalline as observed by X-ray diffraction and high-resolution transmission electron microscopy (TEM). In vitro metabolic toxicity assays using hepatocyte and macrophage cells demonstrate that both IrNPs and iridium(III) chloride are well tolerated at concentrations of up to 10 μM iridium. Furthermore, the IrNPs were assessed in a hemolytic assay and found to have no significant impact on red blood cells when exposed to concentrations up to 100 μM. Overall, these results support the potential for the in vivo application of this nanomaterial.

**Electronic supplementary material:**

The online version of this article (10.1186/s11671-018-2621-3) contains supplementary material, which is available to authorized users.

## Background

Noble metal nanoparticles are a mainstay of emerging nanotechnologies due to their interesting optical, electronic, and surface catalytic properties. In nanomedicine, these unique biomaterials have drawn significant attention due the ability to tailor their biological interactions through surface modifications for a wide range of applications [1]. Gold nanoparticles (AuNPs) have been investigated extensively for sensing and therapeutic applications [2, 3], while other noble metals, including silver, have found niche uses such as anti-microbials [4]. However, nanoparticles composed of platinoid elements, which are commonly employed for their surface catalytic properties [5], have yet to be thoroughly examined for biomedical applications. The exceptional surface stability and known biological compatibility of these elements, as well as their potential novel physical properties on the nanoscale, make them unique alternatives to AuNPs.

High-energy radiation is utilized extensively in medicine including in diagnostic imaging and radiation therapy. Therefore, functional materials that interact with radiation, such as high atomic number and high-density nanoparticles, may improve the performance of these modalities. The majority of the chemical and engineering studies to date have focused on AuNPs to enhance radiation interactions, although bismuth and hafnium have been examined for diagnostic and therapeutic applications respectively [6, 7].

Here, we present a synthetic method to produce iridium nanoparticles (IrNPs), which are predicted to have strong radiation attenuation due to its high density. Iridium is one of the least reactive metals, considered generally biologically compatible, and has an elemental density of 22.56 g/cm^3^ (second only to osmium, which is known to be highly toxic). An isotope of iridium, ^192^Ir, is a commonly used brachytherapy gamma emitter, and part of the success of this material is due to the high density, i.e., the large number of atoms in a small volume of the material. In the current study, we present the synthesis of IrNPs and their in vitro biocompatibility as well as that of iridium ions, which has not previously been evaluated in the selected cell lines. These novel IrNPs have not been readily explored for medical purposes despite the material’s chemical inertness and superior density. Although iridium is a relatively expensive material like other noble metals, its current value as a commodity is approximately three quarters the price of gold and half that of rhodium, making it an interesting economic alternative.

## Methods

### Synthesis of IrNPs

All synthesis reactions were performed at room temperature under aerobic conditions in purified 18 MΩ water. A 20 mM iridium (III) chloride (Acros Organics) stock was prepared by bath sonication and stirred for at least 20 min to generate an optically clear solution. A solution of 1.0 M borane morpholine (Alfa Aesar) was also prepared by bath sonication. For larger scale syntheses of 500 mL total volume, 25 mL iridium (III) chloride solution was used (diluted to 1.0 mM) and 5.0 mL borane morpholine was added (final 10 mM concentration) with rapid stirring. The solution gradually turned from dark brown to black over 30 min. The nanoparticles were allowed to stabilize for at least 60 min. This colloidal solution was directly added to centrifugal spin filters (Amicon Ultra-4, 10k MWCO regenerated cellulose), and the nanoparticles were collected at 4000×*g* and washed in purified water. The nanoparticles were then suspended in water, passed through a syringe filter (Millex-MP 0.22 μm EO), and stored for quantification.

### Nanoparticle Characterization

For X-ray photoelectron spectroscopy (XPS) analysis, nanoparticles were suspended in an equal volume nitric acid, collected by centrifugation in a microcentrifuge tube (5 min, 17 rcf), and suspended in water prior to analysis. Transmission electron microscopy (TEM) was performed on an FEI Tecnai F-20 TEM operating at 200 kV. Purified IrNPs were drop-cast on holey carbon Cu supported TEM grids (Ted Pella) and dried at room temperature overnight. Line diffraction analysis was performed using ImageJ software analysis. For X-ray diffraction (XRD) analysis, concentrated IrNPs were drop-cast on a glass slide and dried at room temperature. XRD data were collected in focused beam (Bragg–Brentano) geometry on a Rigaku Ultima IV X-ray diffraction system using graphite monochromatized Cu Kα radiation. Scans were performed over the angular range 20–80° 2θ at a scan rate of 0.1°/min at room temperature. Dynamic light scattering (DLS) was performed on a Malvern Nano ZSP in disposable polystyrene cuvettes. Nanoparticles were suspended in water, and data is reported as distributed by number. UV-Vis absorbance spectra were collected on a Tecan M200 Pro in a black 96-well plate and a total solution volume of 100 μL. Concentrations of iridium were adjusted to illustrate relative absorbance peaks. XPS analysis was carried out on a PHI Versaprobe II fitted with a hemispherical electron analyzer and aluminum Kɑ (1486.7 eV) X-ray source. Spectrum analysis was performed using the Multipak software suite. Binding energy calibration was performed using the C1s peak at 284.6 eV, and peak fitting was based on asymmetric peaks and an iterated Shirley background, resulting in a chi-squared value of 1.13. Inductively coupled plasma mass spectrometry (ICP-MS) of IrNPs and iridium(III) chloride solution were assessed prior to biological toxicity assays. Fifty microliters of each IrNP solution was digested in 50 μL aqua regia (3:1 M concentrated nitric acid to hydrochloric acid) overnight at 70 °C in a digestion tube. Samples were then diluted in 5.0 mL 1% nitric acid for analysis. ICP-MS was performed on an Agilent 7900 using helium as a collision gas. Calibration curves were prepared using 100–0.1 μg/mL iridium stock solutions (in 1% HCl), and all samples were diluted such that concentrations were measured in the tens of ppb range.

### Cytotoxicity Analysis

HepG2 and J774A.1 cell lines were seeded at 2 × 10^5^ cells per well (100 μL) in a 96-well plate (DMEM with 10% FBS) and allowed to settle for 24 h. Iridium nanoparticles, iridium salt, water, or DMSO was added at 10% volume (10 μL additional volume). Cells were then incubated for 24 or 48 h. For viability analysis, media was removed, and cells were washed once in PBS. One hundred microliters of culture media with 10% Alamar Blue (Thermo Scientific) was incubated with cells for 2 h*.* Media was then re-plated into a black 96-well plate, and fluorescence was read (ex530/em590) on a Tecan M200 Pro. All data were performed in quadruplicate, and experiments were repeated on independent days to confirm general trends. A hemolytic assay was performed as previously reported [8].

## Results

### Iridium Nanoparticle Synthesis and Characterization

**Fig. 1:**
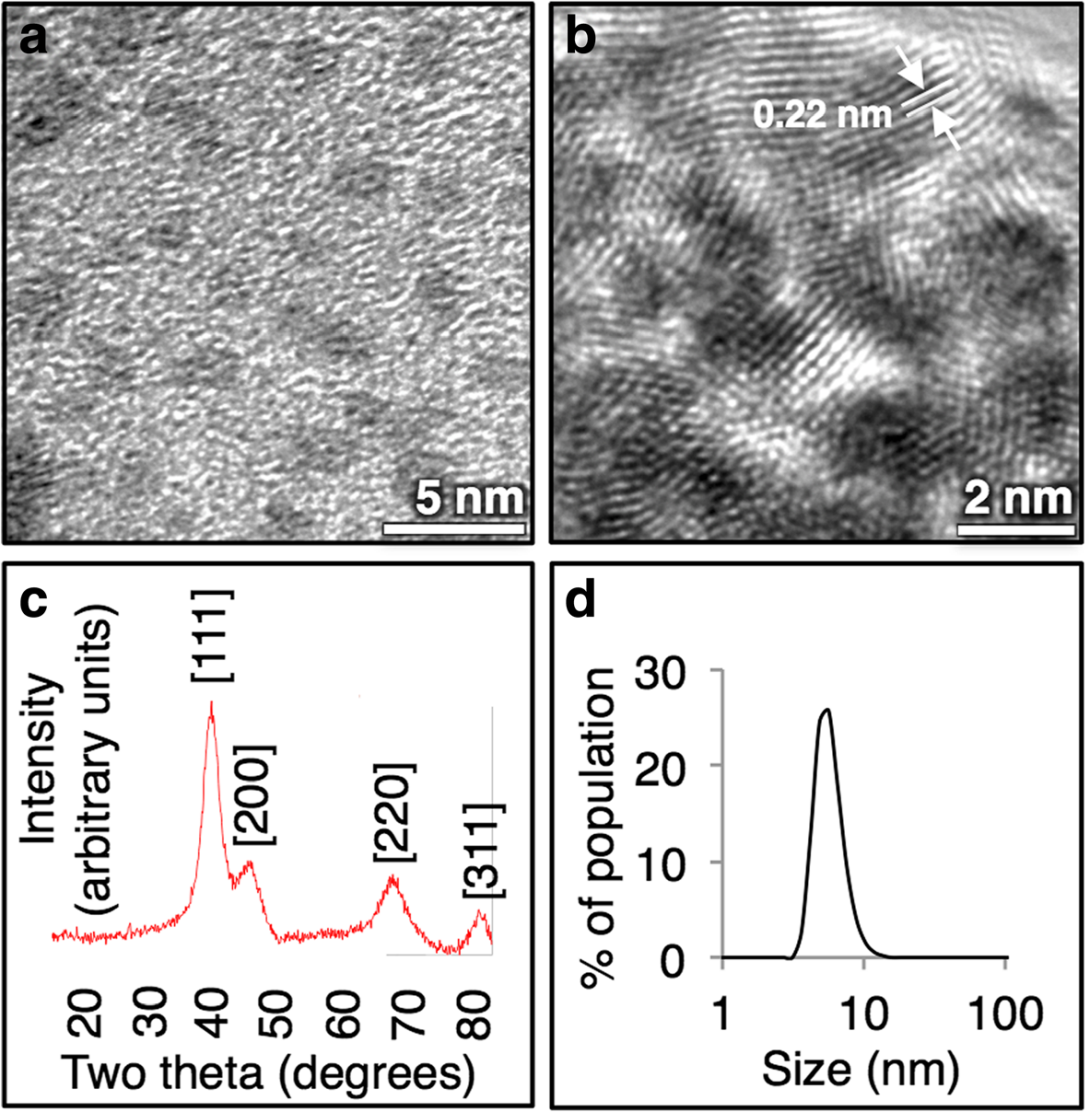
**a** Iridium nanoparticles are 2–3 nm by TEM imaging, with **b** with a highly crystalline lattice parameter. **c** XRD spectrum matches elemental iridium, and **d** particles have a hydrodynamic size of 5 nm in water by DLS

In this synthesis, we form elemental IrNPs from iridium(III) chloride salt by reduction with a 10-fold molar excess of borane morpholine in water. The reaction is readily scalable to multiple liters, and particles are formed at room temperature under aerobic conditions. This synthetic method produces small (2–3 nm) uniform IrNPs (Fig. 1a) with a high degree of crystallinity as observed by high-resolution TEM imaging. Diffraction patterns obtained from TEM further confirm the identity of the nanocrystals, with a line spacing of 0.22 nm that is indicative of the diffraction grating of iridium (Fig. 1b). The X-ray diffraction pattern closely matches that of elemental iridium (PDF Card No.: 9008470, Fig. 1c). As synthesized, IrNPs are colloidally stable in water and remain suspended in solution for several months at room temperature (Fig. 1d).

**Fig. 2:**
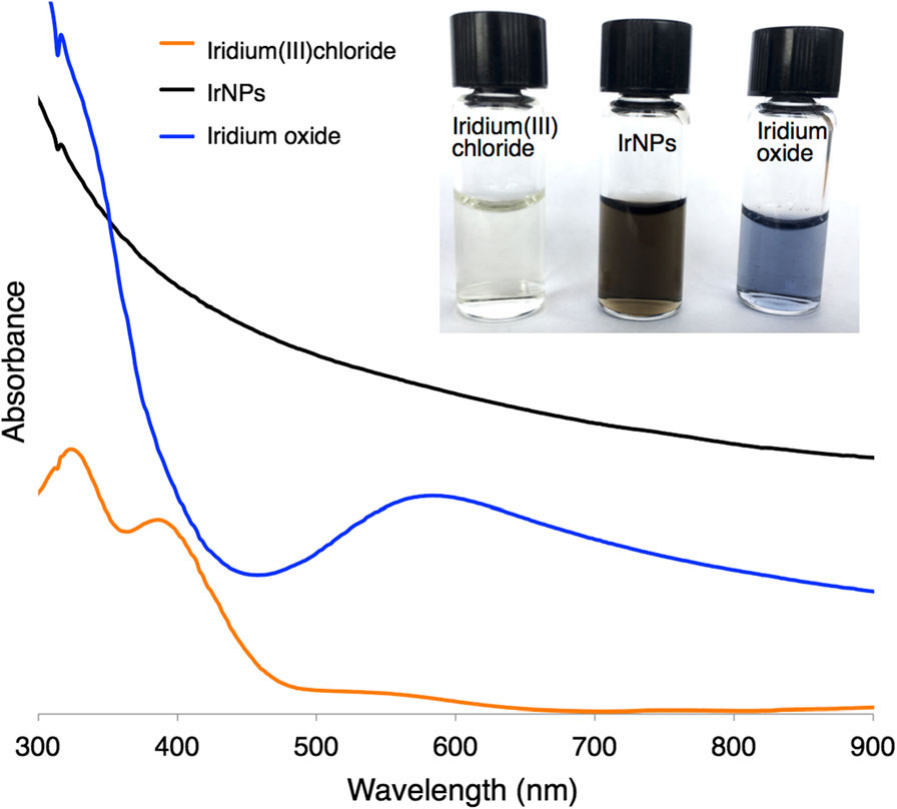
Iridium(III) chloride appears pale yellow with absorbance peaks at 324 and 386 nm. IrNPs are broad-spectrum absorbers and appear black. Iridium oxide (predicted), produced from oxidized IrNPs treated in a basic solution, appears blue-purple purple with an absorbance peak at 584 nm

**Fig. 3:**
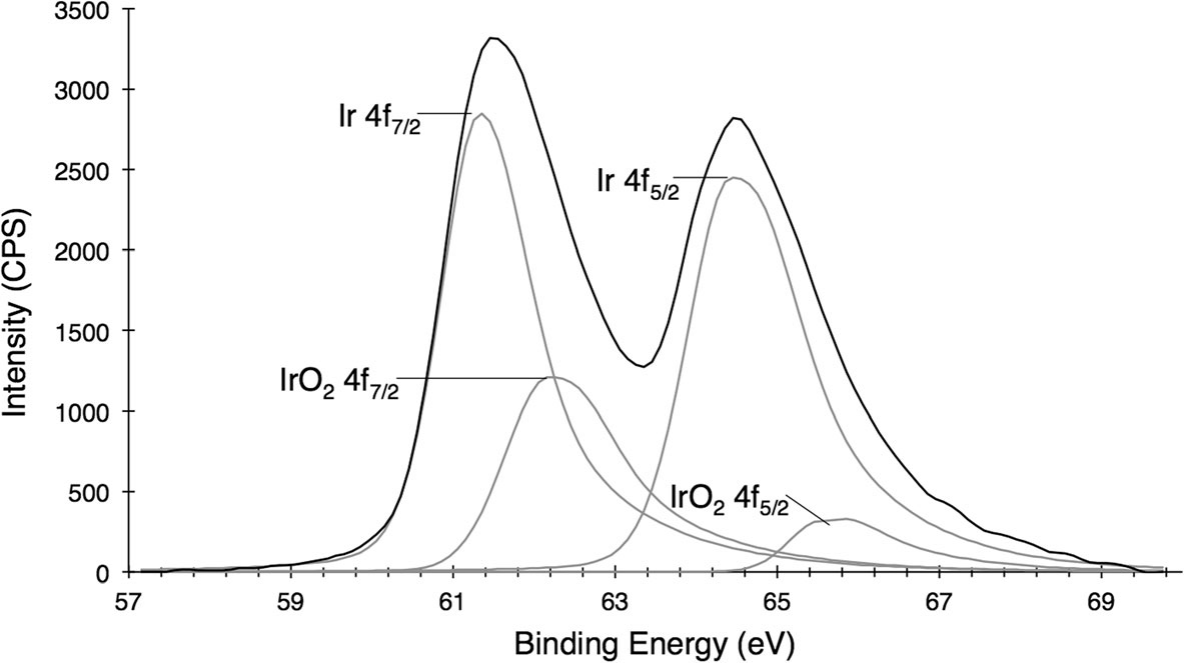
X-ray photoelectron spectroscopy (XPS) of IrNPs a predominantly elemental iridium surface state, with approximately 20% oxide surface contamination

Nanocrystals form over the course of 30 mins as observed by a color change from the light yellow iridium(III) precursor to a dark black nanoparticle solution (Fig. 2). When exposed to a basic environment, these IrNPs form a predicted iridium oxide, which appears blue. Acidic conditions, such as incubation in neat nitric acid, do not appear to impact particle crystallinity or integrity of the material; however, it does induce flocculation and precipitation. In addition, aggregation was also observed in biologically relevant solutions (phosphate buffered saline and tissue culture media) over the course of hours, suggesting that further surface modification will be necessary for future biomedical applications. X-ray photoelectron spectroscopy analysis of the IrNPs rinsed in nitric acid and suspended in water reveals a predominant iridium(0) surface, although peak fitting analysis of data indicates 20% surface oxidation (Fig. 3). No preferred crystallite orientation of particles is observed, either by XRD or by XPS. Alternatively, the introduction of a thiol surfactant to the reaction solution during the synthesis process (prior to nucleation) resulted in inhibition of particle formation.

### Iridium Cytotoxicity

**Fig. 4:**
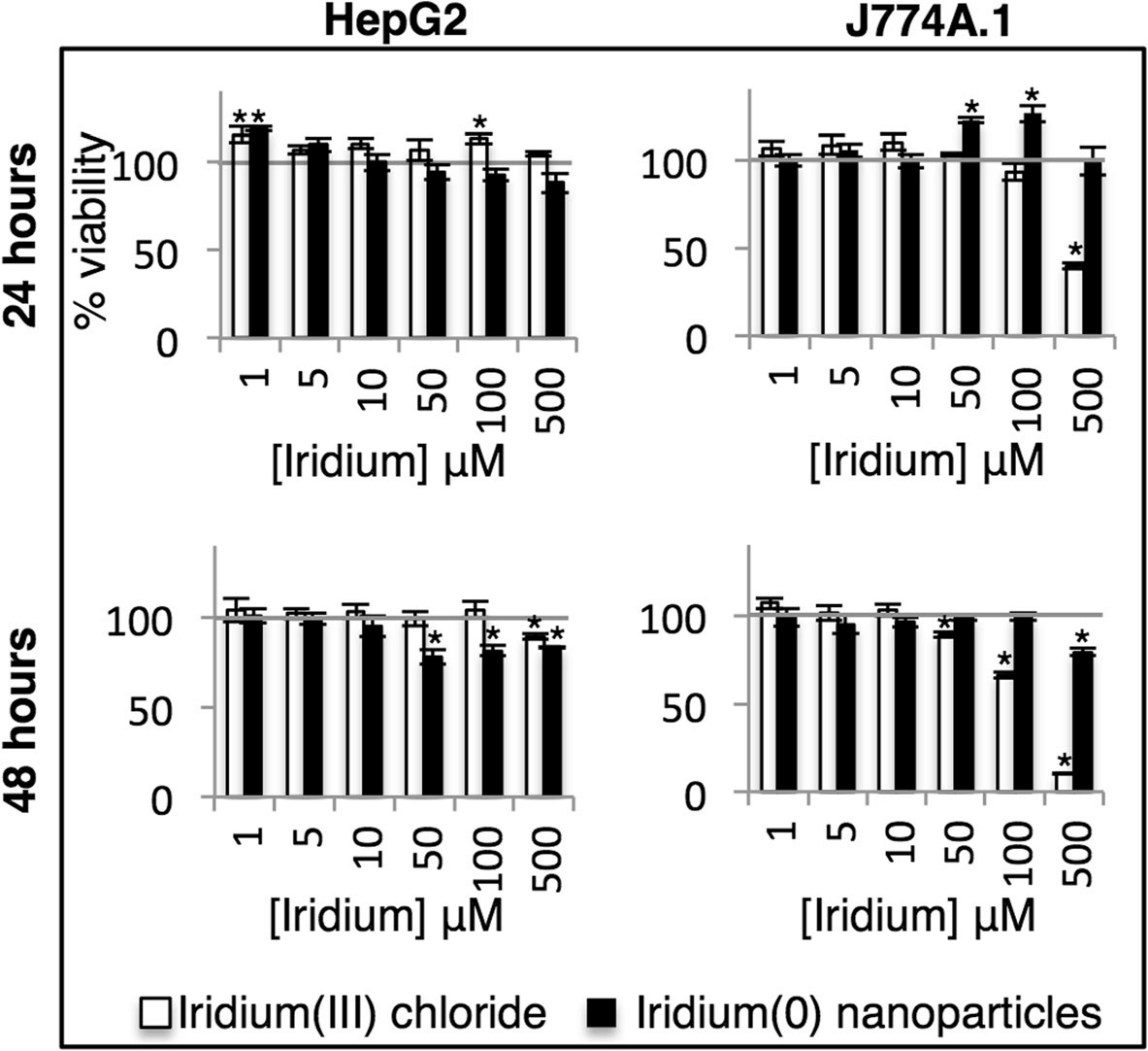
Cellular viability of HepG2 and J774A.1 cells incubated with Ir(0) nanoparticles or Ir(III) salt for 24 or 48 h. *Statistically significant values (*p* < 0.05) relative to untreated cells

We evaluated the in vitro biological compatibility of the uncapped IrNPs and compared this to iridium(III) chloride salt in two types of mammalian cells. HepG2, a hepatocyte carcinoma cell line, was used to evaluate potential toxicity to the liver. J774A.1 macrophage cells were used to evaluate toxicity to the mononuclear phagocytic system. Cells were incubated with IrNPs or iridium(III) chloride (normalized for total concentration of iridium) for 24 or 48 h and washed to remove extracellular iridium, and metabolic activity was evaluated using the Alamar Blue assay (Fig. 4). HepG2 cells show increased metabolic activity in the presence of iridium(III) at 24 h (up to 115% viability), but the response is mitigated by 48 h, with 500 μM iridium(III) reducing viability to 90%. The HepG2 cells had a reduced cellular viability, from 94 to 78% in the presence of 50 μM IrNPs at 24 and 48 h. Interestingly, J774A.1 cells show an increase in metabolic activity in response to IrNPs at a 50-μM concentration with a 122% viability at 24 h; however, after 48 h, normal cellular function was resumed (98% viability), suggesting a transient metabolic stimulation in response to the nanomaterials. J774A.1 cells incubated with 500 μM IrNPs for 24 h show an apparently neutral metabolic response, but the decrease in viability at this concentration after 48 h suggests this is a result of toxicity and metabolic stimulation that appears as a neutral viability response. In addition, we evaluated the in vitro biocompatibility of IrNPs with blood through a hemolytic assay and found IrNPs induced no significant hemolysis when incubated with erythrocytes in PBS at 37 °C for 1 h (Additional file 1: Figure S1).

## Discussion

Various synthetic processes have been examined to produce nanoscale iridium for catalytic applications, including reduction of iridium salts by hydrides and hydrogen gas [9–13], UV and gamma radiation [14–17], and polyol or alcohol reduction [18–20]. However, many of these synthetic methods are designed for integration of iridium onto a substrate or support for chemical reactions and are not compatible with biological applications [21]. Recently, aerosolized ^192^Ir was employed as model nanoscale materials for lung toxicity and was chosen for its exceptional inertness [22, 23]. The primary purpose of these studies was to examine the clearance and translocation of inhaled fine particulates from the lungs; however, it also highlights the biocompatibility of this element.

We evaluated the in vitro biological compatibility of the uncapped IrNPs and compared this to iridium(III) chloride salt in two types of mammalian cells that are expected to accumulate the highest concentrations of injected nanoparticles. Iridium(III) toxicity in J774A.1 cells follows a normal toxicity dose-response curve; 100 μM iridium(III) reduces cellular viability to 93 and 66%, and 500 μM results in a 40 and 10% cellular viability at 24 and 48 h respectively. This data reflect interesting cell-specific response to iridium(0) and iridium(III), and we anticipate further exploring these effects in vivo. Smaller IrNPs and other poorly soluble inorganic nanomaterials are expected to be translocated to the kidney and the liver, with a short temporal residence in the kidneys, and longer residence in the liver, which may further impact cell-specific toxicity profiles. Excretion is expected through the feces for larger iridium particles, although we expect the extremely small size of these IrNPs may be readily filtered through the renal system if colloidal stability in vivo can be maintained [23].

In preparation for in vivo applications, the blood compatibility of the IrNPs was evaluated by a hemolytic assay. Utilizing whole mouse blood, we evaluated the effect of these IrNPs on the rupture of erythrocytes and potential release of hemoglobin. Although in-depth studies of the final surface modified IrNP will need to be evaluated, the current IrNP building blocks do not elicit a detectable hemolytic response until extremely high concentrations (500 μM).

## Conclusions

We conclude from these studies that iridium(0) nanocrystals can be readily synthesized by a simple aqueous borohydride reduction of iridium(III) chloride, which results in 2–3 nm highly crystalline nanoparticles that are colloidally stable in water with an approximately 5 nm hydrodynamic size. During acute exposure, these particles are non-toxic at concentrations up to 50 μM iridium (compared to 10 μM for iridium chloride) in hepatocytes, stimulate metabolic activity in macrophage cells, and do not elicit a hemolytic response at practical concentrations. These ligand-free nanoparticles may serve as building blocks or cores for subsequent surface-modified IrNPs for use in biological and medical applications. Further investigation of the functional properties of these high-density nanomaterials in the presence of X-rays or other radiation presents the opportunity for novel therapeutic and diagnostic agents.

## Additional File

Additional file 1: Hemolytic assay—The blood compatibility of IrNPs (0-500 μM) was evaluated by monitoring hemolysis of red blood cells. No significant hemolytic activity was observed until the highest concentration of 500 μM is reached. Triton-X-100 served as a positive control. (PDF 110 kb)

## Abbreviations

AuNP: Gold nanoparticle
DLS: Dynamic light scattering
ICP-MS: Inductively coupled plasma mass spectrometry
IrNP: Iridium nanoparticle
PDF: Powder diffraction file
UV: Ultraviolet
XPS: X-ray photoelectron spectroscopy
XRD: X-ray diffraction
