# Supplementary material for: Facile Synthesis of Ligand-Free Iridium Nanoparticles and Their In Vitro Biocompatibility
Source: Nanoscale Res Lett. 2018 Jul 13;13:208. doi: 10.1186/s11671-018-2621-3 (PMC6045523; doi:10.1186/s11671-018-2621-3)
Supplement: Supplementary file 1 — Hemolytic assay—The blood compatibility of IrNPs (0-500 μM) was evaluated by monitoring hemolysis of red blood cells. No significant hemolytic activity was observed until the highest concentration of 500 μM is reached. Triton-X-100 served as a positive control. (PDF 110 kb) [file 11671_2018_2621_MOESM1_ESM.pdf]

Facile synthesis of ligand-free iridium nanoparticles and their *in vitro* biocompatibility

Anna L. Brown,[1] Hayden Winter,[2] Andrea M. Goforth,[2] Gaurav Sahay,[1,3] and Conroy Sun[1,4]*

[1] *Department of Pharmaceutical Science, Oregon State University, Portland, OR, USA*
[2] *Department of Chemistry, Portland State University, Portland, OR, USA*
[3] *Department of Biomedical Engineering, Oregon Health and Science University, Portland, OR, USA*
[4] *Department of Radiation Medicine, Oregon Health and Science University, Portland, OR, USA*

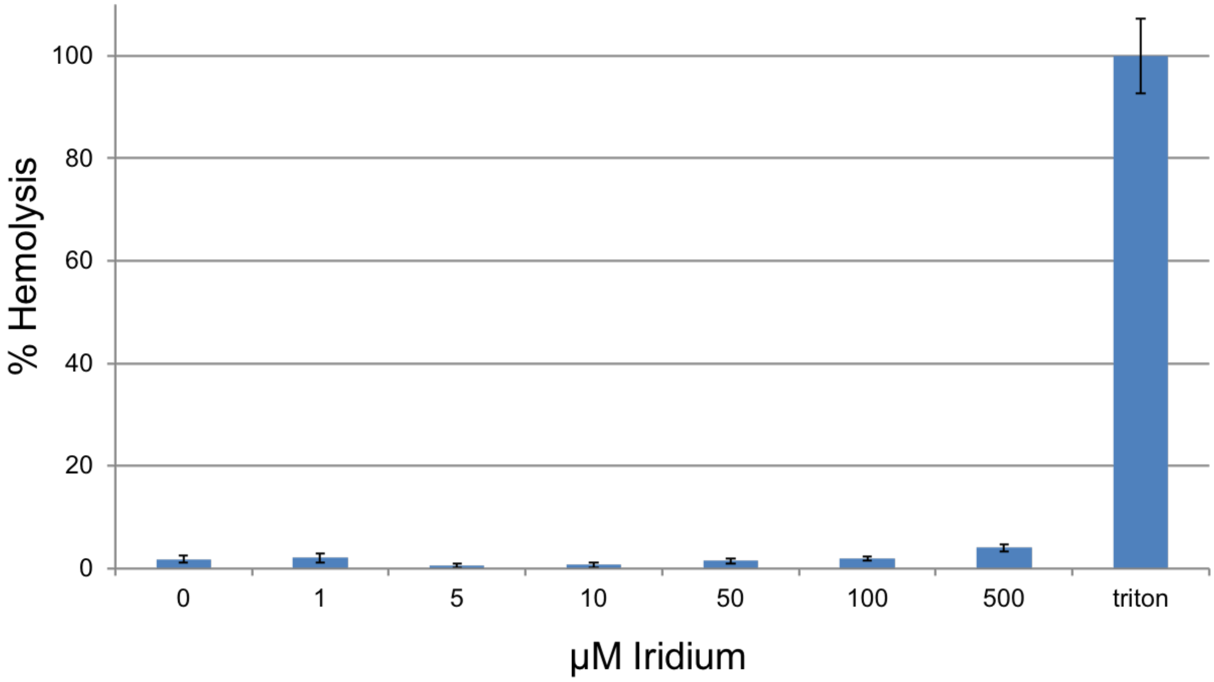

Figure S1 Hemolytic Assay – The blood compatibility of IrNPs (0-500 µM) was evaluated by monitoring hemolysis of red blood cells. No significant hemolytic activity was observed until the highest concentration of 500 µM reached. Triton-X-100 served as a positive control.
